# Antiangiogenic Activity and in Silico Cereblon Binding Analysis of Novel Thalidomide Analogs

**DOI:** 10.3390/molecules25235683

**Published:** 2020-12-02

**Authors:** Megan L. Peach, Shaunna L. Beedie, Cindy H. Chau, Matthew K. Collins, Suzana Markolovic, Weiming Luo, David Tweedie, Christian Steinebach, Nigel H. Greig, Michael Gütschow, Neil Vargesson, Marc C. Nicklaus, William D. Figg

**Affiliations:** 1 Basic Science Program, Chemical Biology Laboratory, Frederick National Laboratory for Cancer Research, National Cancer Institute, Frederick, MD 21701, USA; megan.peach@nih.gov; 2 Molecular Pharmacology Section, Genitourinary Malignancies Branch, Center for Cancer Research, National Cancer Institute, NIH, Bethesda, MD 20892, USA; shaunnabeedie90@gmail.com (S.L.B.); chauc@mail.nih.gov (C.H.C.); collinsmk@nih.gov (M.K.C.); suzana.markolovic@gmail.com (S.M.); 3 School of Medicine, Medical Sciences & Nutrition, Institute of Medical Sciences, University of Aberdeen, Aberdeen AB25 2ZD, UK; n.vargesson@abdn.ac.uk; 4 Drug Design & Development Section, Translational Gerontology Branch, National Institute on Aging, NIH, Baltimore, MD 21224, USA; luowe@grc.nia.nih.gov (W.L.); tweedieda@grc.nia.nih.gov (D.T.); greign@grc.nia.nih.gov (N.H.G.); 5 Pharmaceutical Institute, University of Bonn, 53121 Bonn, Germany; c.steinebach@uni-bonn.de (C.S.); guetschow@uni-bonn.de (M.G.); 6 Chemical Biology Laboratory, Center for Cancer Research, National Cancer Institute, NIH, Frederick, MD 21701, USA; nicklaum@mail.nih.gov

**Keywords:** angiogenesis, cereblon, docking, structure–activity relationships, thalidomide

## Abstract

Due to its antiangiogenic and anti-immunomodulatory activity, thalidomide continues to be of clinical interest despite its teratogenic actions, and efforts to synthesize safer, clinically active thalidomide analogs are continually underway. In this study, a cohort of 27 chemically diverse thalidomide analogs was evaluated for antiangiogenic activity in an ex vivo rat aorta ring assay. The protein cereblon has been identified as the target for thalidomide, and in silico pharmacophore analysis and molecular docking with a crystal structure of human cereblon were used to investigate the cereblon binding abilities of the thalidomide analogs. The results suggest that not all antiangiogenic thalidomide analogs can bind cereblon, and multiple targets and mechanisms of action may be involved.

## 1. Introduction

Despite its teratogenic toxicity, the antiangiogenic and anti-immunoinflammatory efficacy of thalidomide and its analogs have expanded its clinical use in the treatment of Hansen’s disease as well as multiple myeloma and other cancers [1,2,3]. As a result, there are intense efforts to improve the potency of thalidomide while decreasing its toxicity.

Thalidomide was originally believed to be a multitarget drug due to its wide range of biological effects, including reduced TNF-α production, decreased or destabilized COX-2 expression, downregulation of VEGF and FGF, NF-κB inhibition (possibly by suppression of IκB kinase), inhibition of prostaglandin E2 secretion, and α1-acid glycoprotein binding [4,5]. In 2010, however, cereblon was identified as the single target of thalidomide binding, using an affinity purification assay with thalidomide-linked magnetic nanoparticles [6]. Cereblon is the substrate recognition component of a DDB1-CUL4-RBX1 E3 ubiquitin ligase complex. The current model for the bioactivity of thalidomide and its analogs is that binding to cereblon itself induces all downstream effects by triggering the ubiquitin-dependent proteasomal degradation of substrates for the E3 complex [7,8,9].

**Figure 1:**
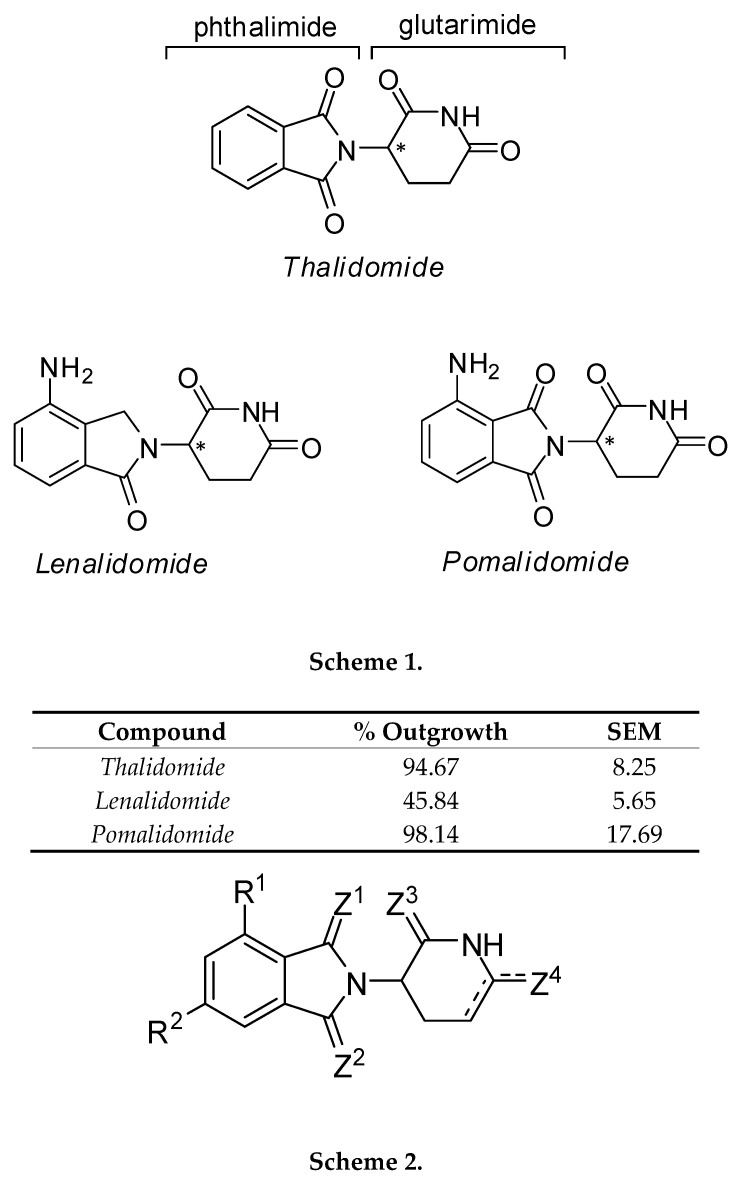

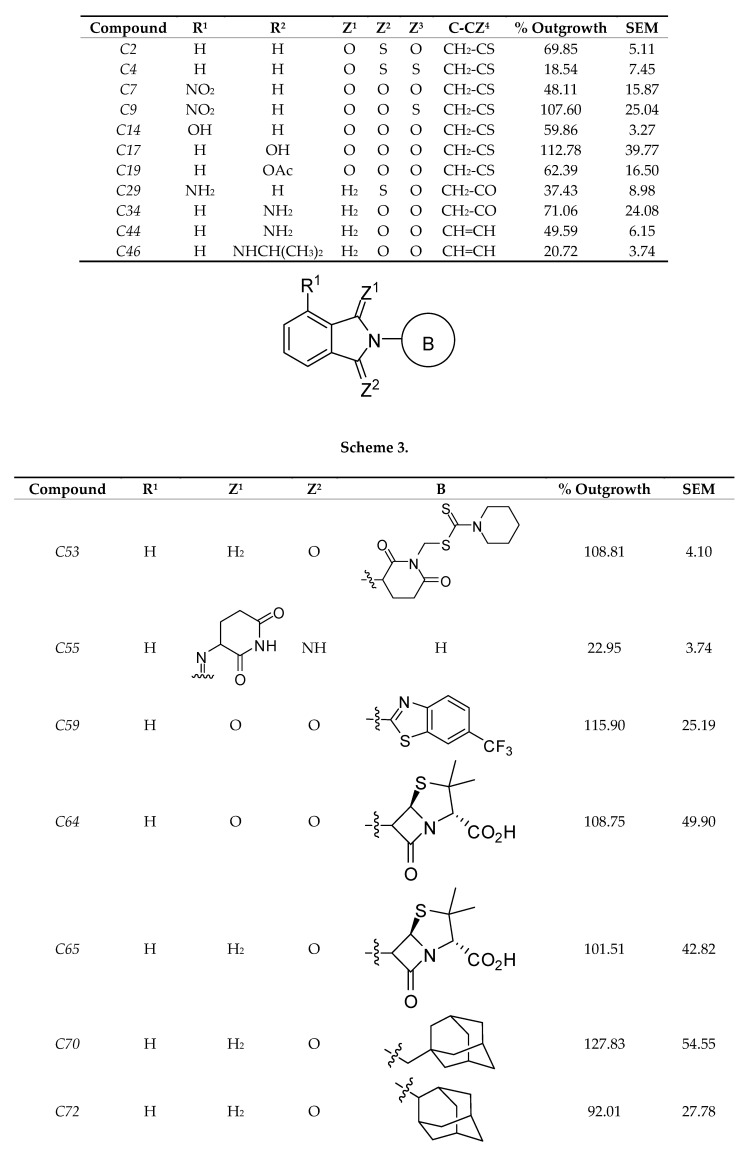

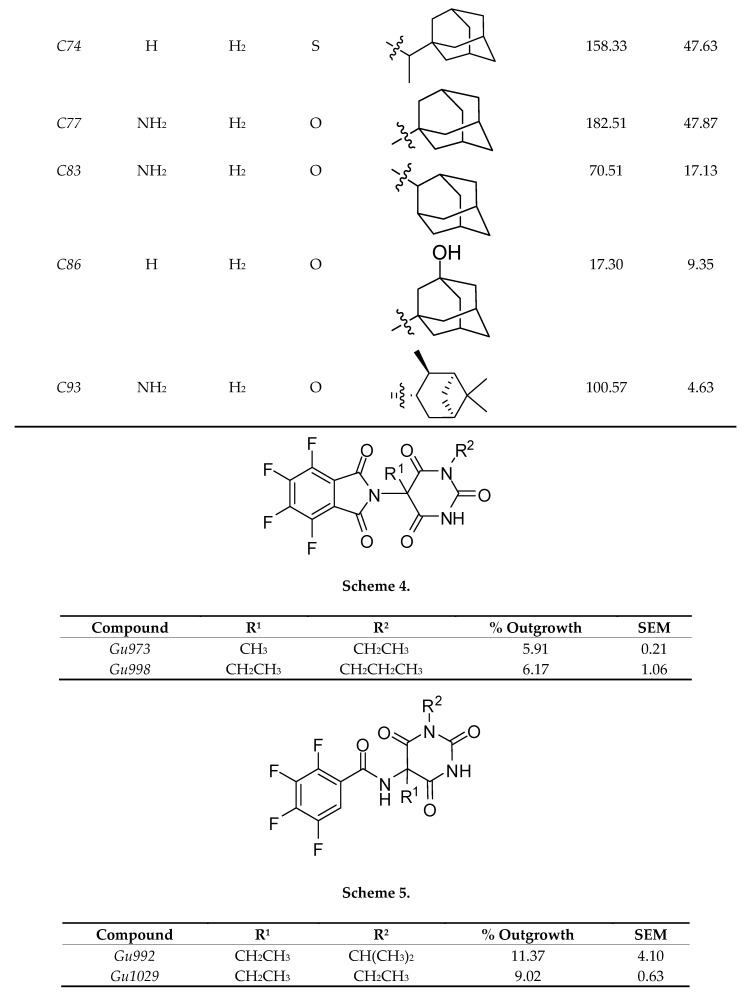
Structures of thalidomide analogs tested and docked. Scheme 1: structures of thalidomide, lenalidomide, and pomalidomide. Scheme 2 comprises compounds where R^1^ is H, NO_2_, NH_2_, or OH; and R^2^ is H, NH_2,_ OH, OAc, or NHCH(CH_3_)_2_; Z^1^ is O or CZ^1^ is CH_2_. Z^3^ is O or S; Z^4^ is O or S, or C-CZ^4^ is CH=CH. Scheme 3 comprises compounds where R^1^ is H or NH_2_; Z^1^ is O or CZ^1^ is CH_2_, or Z^2^ is iminoglutarimide; ring B is substituted glutarimide or a heterocyclic or polycarbocyclic moiety. Schemes 4 and 5 comprise compounds where R^1^ and R^2^ are alkyl groups. Compounds have been previously described: Scheme 2 [18], Scheme 3 [18,19], and Schemes 4 and 5 [20]. Outgrowth from rat aortic rings after a 5-day incubation is given as a percentage of control outgrowth. SEM is the standard error of the mean. A minimum of three rings was used per compound. Asterisk (*) denotes chiral carbon.

The chemical structure of thalidomide consists of two linked rings, a phthalimide and a glutarimide (Figure 1). Recent studies demonstrate that crystal structures of thalidomide, lenalidomide, or pomalidomide binding to cereblon [10,11,12] have shown that this interaction is mediated almost entirely by the glutarimide ring, whereas the phthalimide ring, along with a small surrounding region of the cereblon surface, is involved in binding to neo-substrates for ubiquitination in the ternary complex [13,14]. Thalidomide possesses one chiral center (the C3-carbon atom of the glutarimide ring) and consists of a racemic mixture of two optical isomers, *(S)*- and *(R)*-enantiomers that interconvert under physiological conditions [15]. Structural and biochemical studies established that the *(S)*-enantiomer exhibited a 10-fold stronger binding to cereblon and inhibition of self-ubiquitylation compared to the *(R)*-isomer with the teratogenic effects induced by the *(S)*-isomer [16,17].

Many previous efforts on designing thalidomide analogs to potentiate various biological effects have focused on modifications of the glutarimide ring [21,22,23]. However, since the interaction with cereblon is mediated by the glutarimide ring, it is possible that some of these analogs may not actually bind cereblon and may be exerting their effects by other mechanisms. Such a situation occurred with a series of thalidomide analogs developed by Celgene, initially based on the structure of thalidomide’s glutarimide ring-hydrolysis products [24]. These compounds, in which the glutarimide ring was replaced by a branched 3,4-dialkoxyphenyl-containing moiety, showed much more potent TNF-α inhibition relative to thalidomide. This was discovered to be due to a shift in the mechanism of action to binding and inhibiting phosphodiesterase 4 (PDE4), which elevates cAMP levels and in turn decreases TNF-α. Thalidomide itself does not bind PDE4 [25].

In this work, a cohort of 27 structurally diverse, recently identified thalidomide analogs [19,20] was tested in an ex vivo rat aorta ring (RAR) assay for angiogenesis inhibition. Notably, several of these analogs were found to have antiangiogenic, anti-inflammatory, and/or anticancer activity in in vivo models [18,26]. Given the chemical diversity of these thalidomide analogs, it is unknown which of them could bind to cereblon and which might have other targets. The recent structural studies of cereblon [10] enabled in silico protein docking experiments to analyze the chemical and structural requirements for cereblon binding and investigate their relationship to antiangiogenic activity.

## 2. Results and Discussion

### 2.1. Biological Testing of Thalidomide Analogs

**Figure 2:**
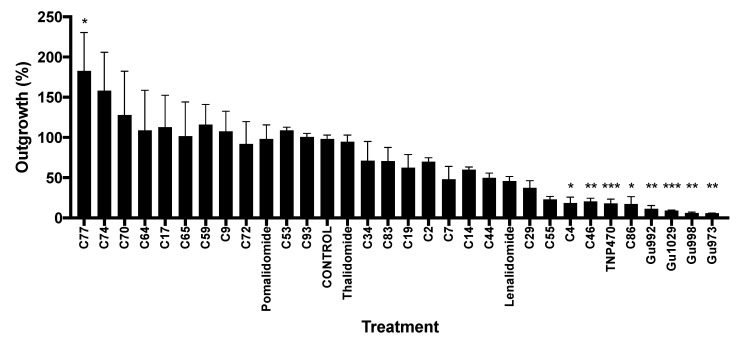
Graphical representation of rat aorta ring (RAR) assay outgrowth. A minimum of three rings were used per treatment. Error bars are SEM. Statistical analysis was performed in Prism and is multiple comparisons (Dunnett’s test) one-way ANOVA (* *p* < 0.05; ** *p* < 0.01; *** *p* < 0.001).

Recent in vitro and in vivo studies have identified the antiangiogenic and therapeutic potential of a novel series of heterocyclic and adamantyl ring-based [18,19] and tetrafluorinated [20,26] thalidomide analogs (Figure 1). To develop a model predictive of antiangiogenic activity, these analogs were screened in an ex vivo angiogenesis model, the rat aorta ring (RAR) assay. We previously demonstrated the RAR assay to be an accurate assessment of antiangiogenic activity, with cytotoxicity having no effect on microvessel outgrowth [27]. This assay, which is more representative than in vitro models and recapitulates the complexities of angiogenesis [28], was selected to assess antiangiogenic activity without in vivo metabolic activation. Using previously described conditions, rat aortic rings treated with TNP-470 (50 μM; positive control) showed little to no outgrowth (17.94% ± 5.42, *n* = 7) compared to vehicle-treated (0.5% DMSO; control) rings (98.05% ± 5.03, *n* = 11). Treatment with thalidomide (50 μM) itself had no effect on outgrowth (94.67% ± 8.25, *n* = 4). While pomalidomide treatment showed a similar response (98.14% ± 17.69, *n* = 4), lenalidomide treatment significantly decreased outgrowth (45.84% ± 5.65, *n* = 4). Compounds *C4*, *C29*, *C46*, *C55*, and *C86* also significantly reduced microvessel outgrowth (*C4* (18.54% ± 7.45, *n* = 4), *C29* (37.43% ± 8.98, *n* = 3), *C46* (20.72% ± 3.74, *n* = 7), *C55* (22.95% ± 3.74, *n* = 3), and *C86* (17.3% ± 9.34, *n* = 3)). The tetrafluorinated compounds were the most potent angiogenic inhibitors in this assay, where the inhibition of outgrowth was indistinguishable from the positive control-treated rings (*Gu973* (5.91% ± 0.21, *n* = 3), *Gu992* (11.37% ± 4.1, *n* = 3), *Gu998* (6.17% ± 1.06, *n* = 3), and *Gu1029* (9.02% ± 0.63, *n* = 4)). Figure 2 summarizes the RAR results of tested analogs.

### 2.2. In Silico Protein Docking Simulation

Biochemical and structural studies have recently identified cereblon as a binding partner for thalidomide [6], and indicated that binding is mediated by the glutarimide ring in a relatively small and hydrophobic binding pocket composed of three tryptophan residues [10,11]. Since the series of thalidomide analogs under investigation here have been mainly designed with modifications to the glutarimide moiety, we investigated whether the cereblon binding pocket is able to accommodate these analogs and the extent to which computationally predicted cereblon binding affinities correlate with antiangiogenic activity in the RAR assay.

The crystal structure of human cereblon in complex with lenalidomide (PDB entry 4TZ4) [10] was selected as the most appropriate for docking, even though it is of relatively low resolution (3.01Å), for two reasons: (1) it is the only available human ortholog structure, and (2) it is the full length cereblon structure rather than the truncated C-terminal cereblon-binding domain. Previous docking studies have shown that the truncated domain, which lacks part of two β-strands and a connecting hairpin loop, is not sufficient for successful docking [29]. In the full-length structure, these strands are stabilized by packing against the N-terminal domain, and the hairpin loop forms part of the binding site [10].

The ligand binding site in cereblon is quite small, and only the glutarimide ring of thalidomide or lenalidomide is buried in the pocket. The phthalimide ring hydrogen bonds to an asparagine residue on the pocket rim, but is otherwise fairly solvent exposed. The glutarimide imide group forms three hydrogen bonds on one side of the pocket, and the aliphatic carbons on the other side of the glutarimide ring are closely packed between three tryptophan residues [10,11]. As a consequence of this close packing, a preliminary docking run (results not shown) found that only compounds with an intact, unmodified glutarimide ring could dock correctly into the binding site. However, studies on the structural dynamics of the bacterial cereblon C-terminal domain have shown large flexibility and even partial unfolding of the domain in the absence of ligand, suggesting that cereblon might be able to adaptively bind native ligands of widely different sizes and chemistry compared to thalidomide [30].

We therefore developed a hybrid ligand-ensemble and induced-fit docking protocol. Ligand-ensemble docking is based on the assumption that ligands structurally similar to the cocrystal ligand should bind in a similar orientation, and even chemically divergent ligands should have some overlap in the types of interactions formed with residues in the binding site. Leveraging this assumption and taking advantage of the existing cocrystal cereblon structures can improve docking results for a series of congeneric analogs [31,32]. An initial docked pose for each thalidomide analog was generated by flexible alignment to the bound lenalidomide crystal ligand in its complex with human cereblon (4TZ4) [10]. Thus, each ligand was initially placed in the same binding mode, aligned via the glutarimide ring (when present) or the phathalimide ring otherwise. In most cases, this initial pose had serious steric clashes with the cereblon binding site, but each protein–ligand complex was then optimized with induced-fit docking.

Induced-fit docking methods model flexibility and conformational changes in the protein binding site in various ways [33]. Here, protein sidechains in the neighborhood of the bound ligand pose were conformationally sampled, repacked, and energy minimized to optimize the binding site fit to the ligand [34,35]. With this protocol, the exact placement of the sidechains in a lower resolution protein structure is less of an issue because specific interactions for each individual ligand are independently optimized while the overall binding mode is maintained. In some cases, repacking of the cereblon binding site to accommodate a larger ligand required one of the tryptophan residues to flip away, out of the binding site, with a high protein conformational energy. We considered this to be evidence that it was not possible for that ligand to fit and bind cereblon. This scenario occurred with compounds having a large or very differently shaped glutarimide ring replacement: *C53*, *C59*, *C64*, and *C65* as well as the methylene-bridged adamantyl compounds *C70* and *C74*.

**Figure 3:**
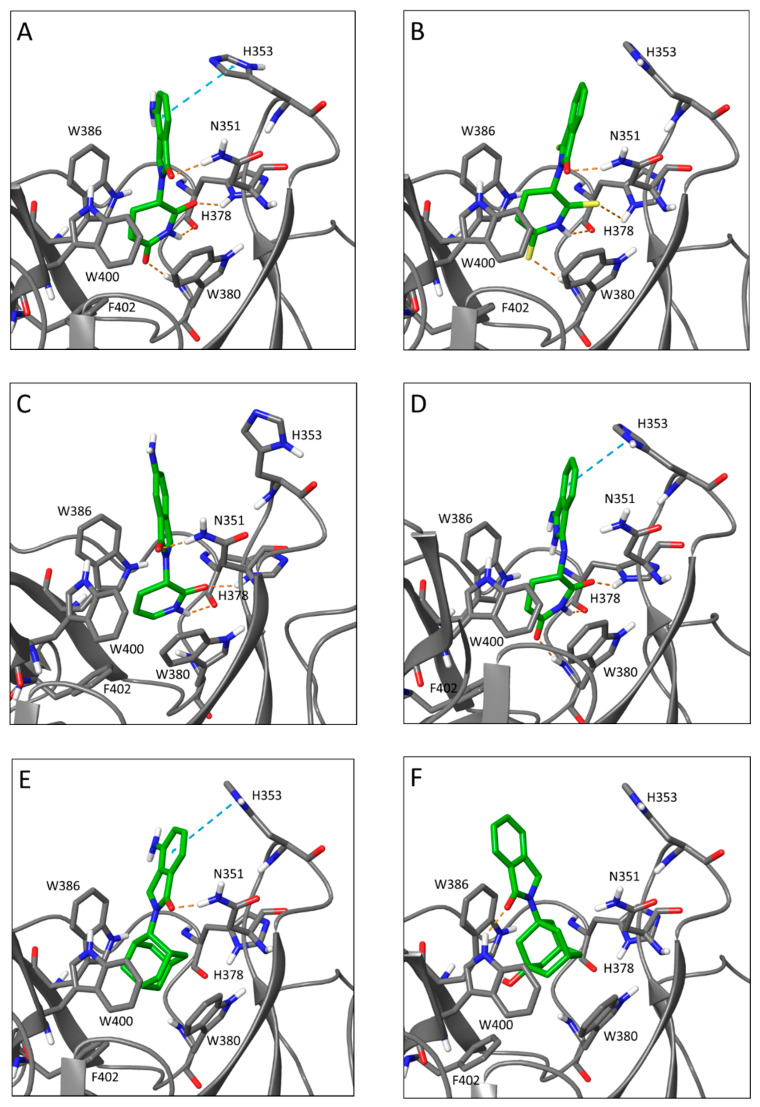

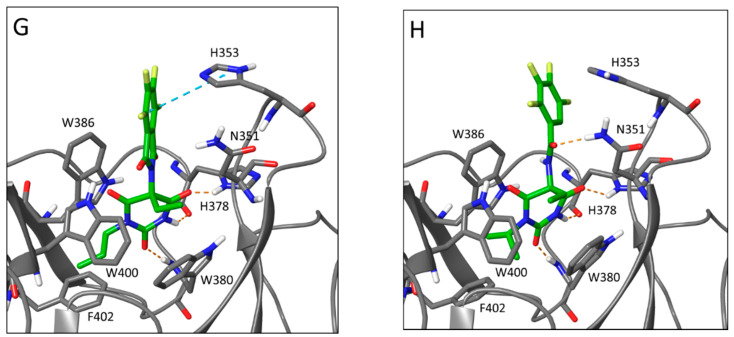
Thalidomide analog docking to cereblon. Hydrogen bonds are indicated by dashed orange lines, and aromatic ring stacking interactions are indicated by dashed light blue lines. (**A**) lenalidomide; (**B**) compound *C4*; (**C**) compound *C44*; (**D**) compound *C55*; (**E**) compound *C83*; (**F**) compound *C86*; (**G**) compound *Gu998*; (**H**) compound *Gu992*. The glutarimide ring makes a set of conserved hydrogen bonds to Trp 380 N and His 378 Nδ and O, and the phthalimide ring can hydrogen bond to Asn 351.

The remaining compounds were able to be accommodated in the cereblon binding site without major sidechain conformational changes and proceeded to the final docking stage, where they were re-docked into their remodeled binding sites and a final induced-fit docking score was calculated as a function of the protein conformational energy and the nonbonded interactions between ligand and protein [35]. Induced fit docking scores are presented in Supplemental Table S1. As expected, thalidomide, pomalidomide, and lenalidomide themselves docked well and scored highly (Figure 3A). The thioxo analogs of thalidomide, *C2*–*C19*, generally fit easily into the cereblon binding site, though they appeared to bind more weakly than compounds with an unmodified glutarimide ring because sulfur is both a weaker hydrogen-bond acceptor and a bulkier atom than oxygen. When present at position Z^3^, the sulfur atom leads to a less preferred accommodation of the ligand than at position Z^4^ (Figure 1), and accordingly compounds *C4* and *C9* have the worst docking scores (Figure 3B). 

Compounds *C44* and *C46*, with an unsaturated planar lactam ring, have a more pronounced tilt angle of the phthalimide skeleton relative to the 3,4-dihydropyridin-2(1*H*)-one moiety. Structures of cereblon complexed with the bound substrates CK1α [14] and GSPT1 [13] show that substrate binding also tilts the isoindolinone substructure in lenalidomide and the analog CC-885 in this same direction, so *C44* and *C46* may have bound conformations preoptimized to facilitate protein–substrate interaction (Figure 3C). Compound *C55*, in which the glutarimide ring has a different attachment geometry, docks well to cereblon. The (*S*)-stereoisomer appears to be preferred. Here, the isoindoline-1,3-diimine is also positioned differently relative to the substrate binding site, and it seems plausible that this may also be favorably affecting substrate binding (Figure 3D), although the exact mechanism for antiangiogenic activity remains to be determined.

None of the adamantane-type compounds (*C70*–*C93*) fit well into the binding site or score well in docking. As mentioned above, the methylene-bridged compounds, *C70* and *C74*, are completely unable to fit. The best-scoring one is the 2-adamantyl derivative *C83* (Figure 3E), and the hydroxylated 1-adamantyl derivative *C86* is barely able to fit into the binding site and shifts the isoindolinone out and away from its position in the other complexes (Figure 3F).

Lastly, the tetrafluorinated compounds, where the glutarimide ring has been replaced with a substituted barbiturate ring, appear to fit well in the binding pocket and score as high as thalidomide itself. Here, however, the (*R*)-stereoisomers are predicted to bind, not the (*S*)-stereoisomers. The barbiturate ring has an additional carbonyl group which remains partially solvent accessible, and the carbonyl groups adjacent to R^1^ and R^2^ (Figure 1) make productive packing interactions with the binding site tryptophan residues (Figure 3G). Opening of the fused five-membered ring and replacing the CO unit, as in compounds *Gu992* and *Gu1029*, slightly shifts the orientation of the tetrafluorobenzoyl ring (Figure 3H). Based on this docking pose, the fluorine atoms would not be involved with cereblon binding, but might affect the interaction with a substrate. 

**Figure 4:**
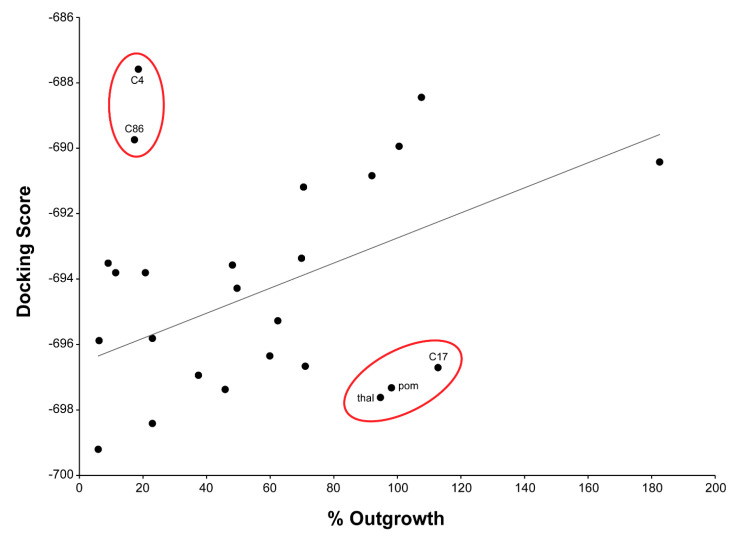
Plot of induced-fit docking score against % outgrowth in the RAR assay. The induced-fit docking score is a function of conformational energy and nonbonded interactions in the receptor–ligand complex. Outliers from the general trend of increased cereblon binding (lower docking score) leading to decreased outgrowth are circled in red.

In Figure 4, the induced-fit docking scores are plotted relative to % inhibition of angiogenesis in the RAR assay. Docking scores are generally at best only loosely correlated with target binding affinity, and it is known that the in vivo effects of thalidomide and its analogs do not necessarily correlate with cereblon binding affinity [36,37], perhaps due to the fact that they are exerting their effects by forming (or inhibiting the formation of) a ternary complex with cereblon, ligand, and substrate. Nevertheless, there was a general trend of increased cereblon binding leading to decreased angiogenic activity.

The outliers are the most interesting compounds to consider here, and these are thalidomide, pomalidomide, and compound *C17*, which are predicted computationally to bind well and yet are inactive in the ex vivo assay. It has been argued that this is because thalidomide requires metabolic activation [38], but it may also be that thalidomide and pomalidomide, with two labile imide substructures, are quite vulnerable to spontaneous nonenzymatic hydrolysis [4,39,40], whereas compounds such as lenalidomide with an isoindolinone ring are hydrolyzed much more slowly and less extensively [41]. The compounds with a thioamide moiety within the five-membered ring (*C29*, *C74*) as well as those bearing a 6-thioxopiperidin-2-one (*C2*, *C7*, *C14*, *C17*, *C19*) or piperidine-2,6-dithione (*C4*, *C9*) are better stabilized and might be less prone to hydrolysis. 

Compound *C17* can be compared to compound *C14*, which differs only in the position of the hydroxyl group on the phthalimide ring (R^2^ vs. R^1^, Figure 1). With the OH group as R^2^, outgrowth is not inhibited at all, whereas with OH as R^1^, outgrowth is reduced to 60%. This effect can also be seen in comparing compound *C34* to lenalidomide, where positioning the amine group either as R^2^ or as R^1^ causes a fairly large shift in activity, from 71% outgrowth in the case of *C34* to 46% outgrowth in the case of lenalidomide.

The other outliers are compounds *C4* and *C86*, which show excellent antiangiogenic activity, yet are not predicted to bind well to cereblon (*C4*) or even to fit into the binding site at all (*C86*). This suggests the possibility that another target or targets could be mediating these effects. We investigated this possibility with a ligand-based pharmacophore analysis. The set of aligned thalidomide analogs was used to develop a set of pharmacophore hypotheses, and multiple potential binding modes were searched for by clustering the active, antiangiogenic compounds according to which pharmacophore hypotheses they matched.

**Figure 5:**
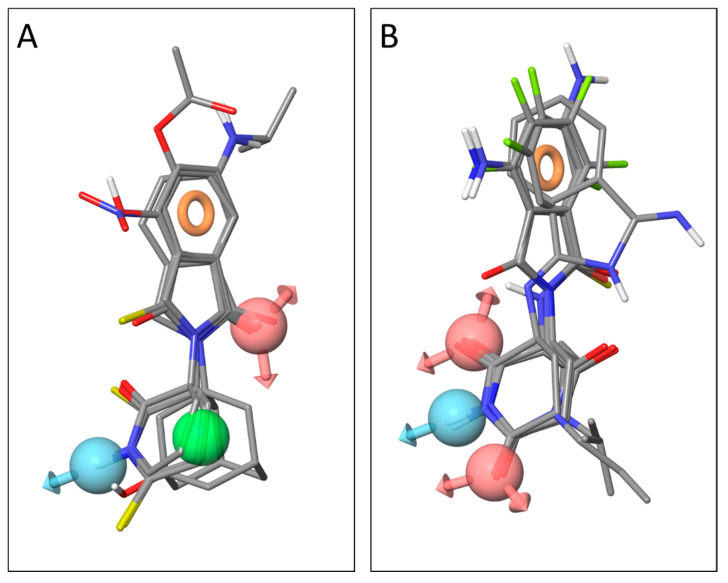
Pharmacophores for two calculated target binding modes. Acceptor site points are colored pink, donors are colored light blue, hydrophobic regions are green, and aromatic rings are orange circles. (**A**) A potential non-cereblon binding pharmacophore for compounds with hydrophobic rings. (**B**) A cereblon-binding pharmacophore for glutarimide-containing compounds.

The pharmacophore clustering revealed two potential binding modes. In this case, rather than different orientations in the same binding site, we suspect similar phthalimide binding sites on different targets. Compounds *C4* and *C86*, along with other thioamide compounds *C2*, *C7*, *C14*, *C19*, and the lactam compounds *C44* and *C46*, can be fit to a pharmacophore with an acceptor feature and an aromatic ring feature on the phthalimide or isoindolinone ring, and a hydrophobic feature along with a donor on the glutarimide or other ring. Thioamides are weak acceptors via their sulfur atoms, but stronger donors than amides, and the hydroxyl group in *C86* can be aligned relatively closely to this donor feature as well (Figure 5A). The other potential binding mode has an aromatic ring feature on the phthalimide and the acceptor-donor-acceptor features on the imide group of the glutarimide ring, which matches lenalidomide, *C29*, *C34*, *C55*, *Gu973*, *Gu998*, *Gu992*, and *Gu1029* (Figure 5B). The docking results suggest that the requirement for a match at the second acceptor site (Z^4^ in the glutarimide, Figure 1) is not necessary for favorable cereblon binding, and thus some compounds can match both pharmacophores.

## 3. Material and Methods

### 3.1. Thalidomide Analogs

A broad series of novel thalidomide-based compounds were synthesized [19,20], dissolved in DMSO, and stored in stock concentration of 10 mM. The chemical structures of lead compounds of interest were confirmed by chemical characterization (purity > 99.5%).

### 3.2. Rat Aorta Ring (RAR) Assay of Angiogenesis

The rat aortic ring assay was performed as previously described [23,26,38]. Briefly, 24-well tissue culture plates were covered with 250 µL of Matrigel (BD Biosciences) and allowed to set for 1 h at room temperature. Six- to eight-week old male Sprague Dawley rats were euthanized, and the descending aortas were dissected and cleaned in EBM media. The aorta was sliced to 1-mm cross-sections, placed on Matrigel-coated wells, and layered with additional Matrigel (250 µL). The rings were allowed to set, after which they were covered with endothelial cell growth media (EGM-II, Lonza, Walkersville, MD, USA) and incubated under 5% CO_2_ at 37 °C overnight. EGM-II consists of endothelial cell basal medium (EBM-II) and endothelial cell growth factors. The next day, media was replaced with EBM-II containing either the vehicle control (0.5% DMSO), 50 μM TNP-470 (a *known angiogenesis inhibitor [42] as the positive control*) or the test compounds at 50 μM. Rings were incubated for 4 days and then imaged on day 5 using an EVOS scope. The experiments were performed in triplicates using aortas from 3-4 different rats. The area of angiogenic sprouting, reported in square pixels, was quantified using Adobe Photoshop. Data was presented as percent growth based on the negative control (vehicle), which was normalized to 100% growth.

### 3.3. Molecular Modeling

All molecular modeling was performed using Schrödinger software (Schrödinger, LLC; New York, NY, USA). version 2017-4. Conformations for the thalidomide analogs were generated in LigPrep using the MMFF94s forcefield [43]. The S chirality was retained for analogs with the same overall scaffold as thalidomide (*C2*–*C53*), and stereoisomers were enumerated for chiral centers in analogs where the scaffold varied (*C55*, *C74*, *Gu973*, *Gu998*, *Gu992*, and *Gu1029*). Two low energy ring conformations were generated for each analog because the glutarimide ring has two possible puckering conformations.

Ligand conformers were aligned flexibly to the crystallized lenalidomide ligand in PDB structure 4TZ4 [10]. For compounds with an unmodified or minimally modified glutarimide ring (*C2*–*C55*), the atoms involved in hydrogen bonding interactions were matched: three carbonyl oxygen acceptors, and the NH donor in the glutarimide ring. For compounds lacking a glutarimide ring (*C59*–*C93*), the phthalimide ring was used for alignment. Both the R and S isomers of the barbiturate ring in compounds *Gu973*, *Gu992*, *Gu998*, and *Gu1029* were aligned to hydrogen bonding atoms of the glutarimide. Upon alignment the isomers differ in whether the nitrogen aligned with the glutarimide donor NH is acyl-substituted.

### 3.4. Induced-Fit Docking

As the ensemble of analog structures was now aligned to the lenalidomide-based pharmacophore in the frame of reference of the 4TZ4 crystal structure, these could now be used as an initial pose input for induced-fit docking. The 4TZ4 structure was prepared for docking [44] by building coordinates for missing residues, setting up zero-order bonds to the zinc atom, deleting all waters, protonating with simplified rules at neutral pH, and optimizing the hydrogen bond network. This was followed by a restrained minimization in the OPLS3 forcefield [45] to a heavy-atom RMSD convergence of 0.3 Å.

This receptor structure, paired with individual ligands from the analog ensemble, underwent Prime protein–ligand complex refinement [34], using the OPLS3 forcefield and the VSGB solvation model [46]. Sidechains with atoms within 5 Å of any of the ensemble members (residues 58–60, 100, 156, 349–357, 374–389, 397, 400–402, 414, and 416 in 4TZ4) were refined with one pass of local optimization sampling, in the default environment with a dielectric of 80.0.

Glide redocking was done with the set of compounds that were able to fit into the cereblon binding site without inducing large-scale unfolding or repacking of the binding side: *C2*–*C46*, *C55*, *C72*, *C77*, *C83*, *C86*, *C93*, *Gu973*, *Gu998*, *Gu992*, and *Gu1029*. Each ligand was redocked into its own induced-fit receptor structure. Glide grids were generated for each receptor structure with default parameters. Glide extra-precision (XP) docking runs sampled nitrogen inversions and ring conformations, penalizing nonpolar amides, with no core matching or constraints [47]. For each ligand, the pharmacophore-aligned conformation was docked as well as the set of original conformers generated by LigPrep. Docked poses were scored using the Prime induced-fit score of XPscore + 0.05 × PrimeEnergy [35].

### 3.5. Pharmacophore Analysis

A pharmacophore model was developed in Phase [48] based on the set of prealigned thalidomide analogs that were used for docking. Active compounds were defined as those with less than 75% outgrowth in the RAR assay. Pharmacophore hypotheses were required to match at least 50% of actives, and to have 3-5 features. Default feature types were used. Since as expected, no single hypothesis matched all the actives, the “Detect Binding Modes” tool was used to perform hierarchical clustering on both the active compounds and the hypotheses, each represented by bit strings. Clustering into 2, 3, or 4 possible binding modes was tested, and results were that active compounds could be consistently grouped into matches with two binding mode pharmacophore hypotheses.

## 4. Conclusions

As a prodrug, thalidomide is appreciably more active in vivo than conveyed by in vitro data [23,38]. Given that thalidomide’s activity is in part due to its biologically active hydroxylated metabolite, it is of interest to identify the physicochemical features of potent thalidomide analogs that do not require hepatic activity. To identify such features, 27 chemically diverse thalidomide analogs were screened in the rat aorta ring (RAR) assay; 16 of these compounds exhibited inhibition of microvessel outgrowth, with seven of these being statistically significant (compounds *C4*, *C46*, *C86*, *Gu973*, *Gu992*, *Gu998*, and *Gu1029*; Figure 2). Here we analyzed the structural requirements for activity in this set of thalidomide analogs.

Molecular modeling approaches have previously been used to examine the associations between the chemical structures of thalidomide analogs and their biological activities. A previous 3D-quantitive structure–activity relationship (3D-QSAR) study on thalidomide analogs used comparative molecular field analysis (CoMFA) and comparative molecular similarity indices analysis (CoMSIA) procedures to identify structural components responsible for antiangiogenic activity [49]. Within this study, 29 structurally related thalidomide analogs were first examined for antiangiogenic activity in the RAR assay, and inhibition values were then used as the basis for CoMFA and CoMSIA analyses. These 3D-QSAR studies identified physicochemical features that affected antiangiogenic activity. In particular, perpendicular alignment of the glutarimide ring to the phthalimido ring, hydrophobicity, and steric bulk around the phthalimido ring, and hydrophilic regions within the side chain were found to promote activity; conversely, steric bulk and hydrogen bond donor groups around the glutarimide ring were identified to decrease activity [49]. These models offered insight into the structural requirements for antiangiogenic activity, speculative knowledge of the binding partner(s), and aid in the design of new thalidomide analogs [20,50,51,52]. Similar CoMFA and CoMSIA approaches were applied to identify trends in the properties of thalidomide analogs with anti-inflammatory activity [53].

3D-QSAR approaches have the fundamental baseline assumption that all compounds are binding to the same target and making the same set of interactions, and further assumes that the correct alignment and superposition of the compounds can be correctly deduced [54,55]. However, trends in the antiangiogenic and anti-inflammatory potencies have led to speculation that more than one target may be mediating the effects of these thalidomide analogs [20,26]. Recently, pull-down assays and X-ray crystallography studies have shown that the target of thalidomide and its close analogs lenalidomide and pomalidomide is the E3 protein ligase complex component cereblon [6,10,11]. Protein docking of analogs can help elucidate if and how they interact with a known target of the parent compound. Using a crystal structure of human cereblon [10], we conducted in silico docking simulations to identify which compounds in the set of 27 thalidomide analogs are likely to bind to cereblon and to gain insight into their binding modes and predicted binding affinities. The small and closely packed nature of the cereblon binding pocket required the development of a hybrid ligand-ensemble and induced-fit docking protocol. This ensured that all compounds could be analyzed in the context of a similar binding mode, as expected for close analogs, and offered a feasible approach to the conformational flexibility of the binding site.

Some structure–activity relationships could be observed directly from the RAR assay results. For example, all compounds with thalidomide-like glutarimide rings (*C29*, *C34*, *C55*) were able to reduce microvessel outgrowth (Figure 2). Of particular interest, the glutarimide of *C55* is intact but is incorporated at position Z^1^ (Figure 1) of an isoindoline-1,3-diimine structure instead of substituting the phthalimide nitrogen (Figure 3D). When treated with *C55*, RAR microvessel outgrowth was reduced to 22.95% (±3.74), suggesting that the functional glutarimide moiety is sufficient for activity, and that modifications of the phthalimide are probably tolerated for cereblon binding. The glutarimide nitrogen of *C53* is substituted with a space-filling residue (Figure 1). This compound did not show antiangiogenic activity in the RAR assay and was not predicted by docking to bind to cereblon.

Previous studies have shown that thalidomide analogs without an intact glutarimide ring do not exhibit antiangiogenic activity in the RAR assay [49]. From the RAR assay data, it appears that other substructures can also be active. Docking results suggest that barbiturate moieties, as well as lactams and thiolactams, might fit into the cereblon binding site. However, compounds *C4* and *C9* bearing a piperidine-2,6-dithione, i.e., a doubly thionated glutarimide, along with compounds with large bicyclo or adamantyl rings (Figure 3E,F) were not predicted to be able to bind cereblon well. A recent paper that systematically characterized the cereblon binding abilities of probe compounds with a variety of 5- and 6-membered rings, using FRET pairing to the binding site tryptophans in a bacterial single-domain homolog of cereblon, found that barbiturate rings, as well as some but not all thiocarbonyl-containing 5-membered rings, did not bind cereblon [56]. This suggests that our induced-fit docking, which was designed to be as generous as possible in accommodating nonglutarimide rings into the binding site, may in fact be too lenient, and thus that even more of the thalidomide analogs examined here may be exerting antiangiogenic effects via a different mechanism.

A pharmacophore analysis suggested that the strongly antiangiogenic, hydrophobic compounds *C4* and *C86* are likely to have another target. In fact, eight of the 13 nonfluorinated compounds with antiangiogenic activity in the RAR assay fit this potential target pharmacophore, although some of these compounds are also predicted to bind cereblon. *C4* and *C86* contain a piperidine-2,6-dithione and an adamantane group, respectively, in place of the glutarimide ring. Interestingly, both adamantane and thioamide derivatives have been reported as ion channel inhibitors [57,58,59]. Adamantane derivatives are largely used as antivirals [60] and in the treatment of Parkinson’s disease by indirect modulation of dopaminergic transmission [61].

All the tetrafluorinated thalidomide analogs (*Gu973*, *Gu992*, *Gu998*, and *Gu1029*) vastly reduced microvessel outgrowth and were predicted to bind cereblon as strongly as thalidomide via their alkylated barbiturate rings. Differences in the barbiturate ring puckering vs. glutarimide ring might shift the position of the phthalimide ring slightly toward the outside of the cereblon binding site (Figure 3G), and this difference might be magnified with the tetrafluorobenzamide derivatives *Gu992* and *Gu1029* (Figure 3H). In addition to this, the polyfluorination of the phenyl ring will nearly invert its quadrupole moment and strongly affect its intermolecular interactions [62]. This might suggest that cereblon complexes with tetrafluorinated analogs catalyze the ubiquitination of a new substrate or attract a new binding partner to inhibit angiogenesis by some other mechanism. A second possibility is that the tetrafluorinated analogs have a second target, specific to the fluorobenzene substructure. This is supported by studies with alkyl substituted tetrafluoro-phthalimides and tetrafluoro-benzamides that also show moderate antiangiogenic activity [63].

Overall, the in silico cereblon docking experiments suggest that cereblon binding alone does not account for the antiangiogenic activity of all the tested thalidomide analogs. Our recent findings demonstrate that loss of cereblon does not prevent thalidomide-induced antiangiogenesis, though downstream cereblon targets are upregulated [64]. Thalidomide itself appears to have one target [6], but analogs and metabolites of thalidomide could have multiple targets. Previous theories on thalidomide’s mechanism of action as a multitarget drug [22,65] have in a way become a self-fulfilling prophecy as modifications to the glutarimide moiety, which is directly involved in cereblon-binding of thalidomide, can lead to new target activities. The phthalimide moiety of thalidomide (and the similar isoindolinone moiety of lenalidomide) have been described as privileged scaffolds that can modulate many targets [66,67].

Though structure-based drug design has its limitations, computational modeling remains a powerful tool for preliminary drug screening and the design of new lead series. The herein described modeling studies must now be combined with the experimental determination of the affinity of this type of ligand to cereblon. Corresponding data will enable future work to identify thalidomide analogs that promote anti-inflammatory and antiangiogenic activity while reducing side effects and teratogenic activity, with the aim of making safer, more potent compounds available for clinical use.

## Supplementary Materials

The following are available online, Table S1: Induced-fit docking scores for compounds able to fit in the cereblon binding site.
