# Supplementary material for: Antiangiogenic Activity and in Silico Cereblon Binding Analysis of Novel Thalidomide Analogs
Source: Molecules. 2020 Dec 2;25(23):5683. doi: 10.3390/molecules25235683 (PMC7730988; doi:10.3390/molecules25235683)
Supplement: Supplementary file 1 [file molecules-25-05683-s001.pdf]

Antiangiogenic Activity and in Silico Cereblon Binding Analysis of Novel Thalidomide Analogs

Megan L. Peach [1], Shaunna L. Beedie [2,3], Cindy H. Chau [2], Matthew K. Collins [2], Suzana Markolovic [2], Weiming Luo [4], David Tweedie [4], Christian Steinebach [5], Nigel H. Greig [4], Michael Gütschow [5], Neil Vargesson [3], Marc C. Nicklaus [6] and William D. Figg [2,*]

[1] Basic Science Program, Chemical Biology Laboratory, Frederick National Laboratory for Cancer Research, National Cancer Institute, Frederick, MD 21701, USA; megan.peach@nih.gov

[2] Molecular Pharmacology Section, Genitourinary Malignancies Branch, Center for Cancer Research, National Cancer Institute, NIH, Bethesda, MD 20892, USA; shaunnabeedie90@gmail.com (S.L.B.); chauc@mail.nih.gov (C.H.C.); collinsmk@nih.gov (M.K.C.); suzana.markolovic@gmail.com (S.M.);

[3] School of Medicine, Medical Sciences & Nutrition, Institute of Medical Sciences, University of Aberdeen, Aberdeen AB25 2ZD, UK; n.vargesson@abdn.ac.uk

[4] Drug Design & Development Section, Translational Gerontology Branch, National Institute on Aging, NIH, Baltimore, MD 21224, USA; luowe@grc.nia.nih.gov (W.L.); tweedieda@grc.nia.nih.gov (D.T.); greign@grc.nia.nih.gov (N.H.G.)

[5] Pharmaceutical Institute, University of Bonn, 53121 Bonn, Germany; c.steinebach@uni-bonn.de (C.S.); guetschow@uni-bonn.de (M.G.)

[6] Chemical Biology Laboratory, Center for Cancer Research, National Cancer Institute, NIH, Frederick, MD 21701, USA; nicklaum@mail.nih.gov

[*] Correspondence: figgw@mail.nih.gov; Tel.: +1-240-760-6179; Fax: +1-240-858-3020

Received: 18 September 2020; Accepted: 27 November 2020; Published: 2 December 2020

Table S1. Induced-fit docking scores for compounds able to fit in the cereblon binding site. The IFD score is calculated as XP score + (0.05 × Prime energy).

	**% growth**	**Prime energy**	**Prime × 0.05**	**XP score**	**IFD score**
len	45.84	−13693.4	−684.670	−12.703	−697.373
thal	94.67	−13693.4	−684.670	−12.949	−697.619
pom	98.14	−13693.4	−684.670	−12.655	−697.325
C2	69.85	−13669.9	−683.495	−9.868	−693.363
C4	18.54	−13621.2	−681.060	−6.528	−687.588
C7	48.11	−13669.9	−683.495	−10.076	−693.571
C9	107.6	−13632.8	−681.640	−6.804	−688.444
C14	59.86	−13695.6	−684.780	−11.567	−696.347
C17	112.78	−13698.6	−684.930	−11.774	−696.704
C19	62.39	−13699.4	−684.970	−10.303	−695.273
C29	37.43	−13707.6	−685.380	−11.560	−696.94
C34	71.06	−13695.5	−684.775	−11.887	−696.662
C44	49.59	−13666.8	−683.340	−10.942	−694.282
C46	20.72	−13677.0	−683.850	−9.956	−693.806
C55 R	22.95	−13722.3	−686.115	−9.696	−695.811
C55 S	22.95	−13722.3	−686.115	−12.298	−698.413
C72	92.01	−13615.7	−680.785	−10.053	−690.838
C77	182.51	−13615.7	−680.785	−9.638	−690.423
C83	70.51	−13615.7	−680.785	−10.402	−691.187
C86	17.3	−13633.5	−681.675	−8.068	−689.743
C93	100.57	−13623.8	−681.190	−8.753	−689.943
Gu973	5.91	−13695.9	−684.795	−14.407	−699.202
Gu998	6.17	−13666.4	−683.320	−12.561	−695.881
Gu992	11.37	−13629.8	−681.490	−12.316	−693.806
Gu1029	9.02	−13629.8	−681.490	−12.022	−693.512
